# Impact of Animal Encounter Modality and Species on Zoo Visitor Knowledge, Concern, and Conservation Intent

**DOI:** 10.1002/zoo.70023

**Published:** 2025-09-15

**Authors:** Lisa P. Barrett, Rebecca J. Snyder

**Affiliations:** ^1^ Oklahoma City Zoo and Botanical Garden Oklahoma City Oklahoma USA; ^2^ Center for the Integrative Study of Animal Behavior Indiana University Bloomington Bloomington Indiana USA

**Keywords:** animal–visitor interactions, cognition, elephant, stingray, zoo

## Abstract

Zoos fill an important role in connecting humans with nature, especially given an increasing rate of both urbanization and biodiversity loss. With the advent of new technologies, however, there are many alternatives to experiencing biodiversity firsthand. We tested if the type of animal encounter at a zoo (in‐person animal viewing without touch, in‐person animal viewing with touch, or video‐recorded animal viewing) and/or animal species (elephant or stingray) affected zoo visitor knowledge, emotional affect, empathic concern, and/or conservation intent. A total of 300 zoo members were randomly assigned to 6 different animal encounter conditions. We found that participants who viewed video‐recordings of animals had significantly lower scores on all outcomes, except knowledge, than those who viewed animals in‐person. There were no significant differences between in‐person animal viewing without touch and in‐person animal viewing with touch. Moreover, we found that stingray participants had lower scores on all outcomes compared to elephant participants. We suggest extensions of this study and discuss potential implications for the future role of zoos.

## Summary

- We provided elephant and stingray encounters in video, in‐person no‐touch, and in‐person touch formats for zoo visitors.
- For affect, concern, and conservation intent, participants in the video condition had significantly lower scores than those in the in‐person touch and no‐touch conditions.
- For knowledge retention, there were no significant differences in condition, but knowledge retention was higher in the video condition for elephant participants.
- Stingray participants had lower knowledge, emotional affect, empathic concern, and conservation intent scores than elephant participants.

## Graphical abstract

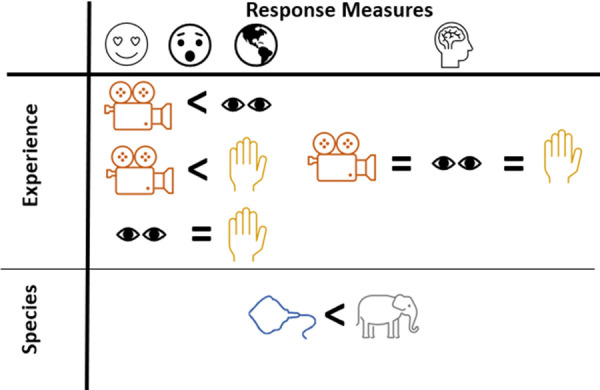

## 1 Introduction

Humankind's disconnect from nature is thought to contribute to a host of social and environmental problems, ranging from ill health in individuals, to community challenges in ensuring sustainability of natural resources, to climate change and the extinction crisis (reviewed in Ives et al. 2018). Exposure to nature is thus viewed by some as a treatment to restore connection with nature. Furthermore, restoring this connection with nature has the potential not only to improve individual wellness, but also to engage societies in solving broader environmental problems, including climate change, pollution, and habitat degradation (Ives et al. 2018). More work is necessary, however, to identify ideal frequency/duration of contact and type of nature connection (i.e., “dose of nature,” Shanahan et al. 2016) needed to effect meaningful change without compromising animal well‐being (Perdue and Maple 2024).

Accredited zoos and aquariums assert that displaying living animals helps connect their visitors to animals specifically and to biodiversity and nature more broadly (Barongi et al. 2015). These organizations also strive to leverage that connection to generate a hierarchy of emotions (i.e., concern, caring, empathy) with the ultimate aim to inspire their visitors to take conservation action (Barongi et al. 2015; Nageotte et al. 2024; Kreger and Mench 1995). This is an ambitious target involving constructs that are challenging to measure with an expansive end goal. Some critics argue, however, that zoos and aquariums are no longer crucial (e.g., Spooner et al. 2023); after all, most people can easily access facts, photos, and videos of nearly every known animal through media (Brossard 2013).

To foster connection with animals, some zoos and aquariums offer behind‐the‐scenes tours of animal facilities, including experiences where visitors meet animals in an “up close and personal” setting (D'Cruze et al. 2019). These experiences sometimes include a touch‐the‐animal component, which may be an especially meaningful way to evoke positive feelings in visitors and a desire to contribute to conservation efforts for the species. However, there is limited empirical evidence on how these types of encounters affect visitors during or after the experience. Some research suggests that zoo visitors who view active animals experience positive affect (e.g., Luebke et al. 2016) and may be more likely to support conservation of wild counterparts (e.g., Hacker and Miller 2016; Skibins et al. 2013), but it remains unclear which aspects of these encounters are most important. For instance, visitors who watched a *video* recording of a polar bear demonstration did not experience increased knowledge retention, empathic concern, and conservation intent compared to visitors who viewed a *live* polar bear demonstration, suggesting that the *live* demonstration was more meaningful than watching a *video* of an animal (Miller et al. 2020).

More recently, Fischer et al. (2024) compared visitors' knowledge gain and attitudes toward zoos before and after different types of human–animal interactions: presentation with live animal present, video of a recorded presentation with live animal present, and a control of a video‐recorded presentation with no animal present, each with four different ambassador animal species. Fischer et al. (2024) found increases in positive emotional experience and empathy for visitors viewing *live* cheetahs and penguins (but not tortoises or sloths) compared to those who viewed a *video* experience. Furthermore, Lacinak (2024) highlighted the inability to touch the animals as visitors' least liked aspect of their encounters with elephants. On the other hand, some studies have found that close proximity to/contact with the animal is *not* critical for meaningful experience‐making (Priestley et al. 2023; Collins et al. 2021; Basden 2022; Howell et al. 2019) and might even distract from knowledge gain (e.g., cheetahs, Whitehouse‐Tedd et al. 2022). For example, Priestley et al. (2023) and Collins et al. (2021) found that visitors who observed animals interacting with enrichment (and without touching the animals) experienced positive emotions and expressed intent to help conserve animals. Moreover, when Howell et al. (2019) surveyed guests about which animals they “felt most connected with,” close proximity to the animal was not significantly related to connection.

Aspects other than proximity to the animals have also been found to affect visitor knowledge, attitudes, and conservation intent. For example, previous research showed that visitors who interacted with zoo staff sharing information about an animal in their care (without close proximity to the animal itself) reported positive attitudes, increased knowledge, and increased engagement in conservation behaviors (reviewed in Priestley et al. 2023). In addition, familiarity with or perceived charisma of the species affects visitors' likelihood to donate to conservation efforts (e.g., Colléony et al. 2017). Thus, more familiar, charismatic species may elicit greater empathy from visitors (Young et al. 2018). George and Cole (2018) found greatest knowledge gain in postsecondary students who viewed a live exotic animal (e.g., cheetah, penguin) compared to students who viewed a live companion or agricultural animal. Additionally, Skibins et al. (2017) found that relatability and conservation status were important factors influencing conservation caring. Given the various factors affecting visitor outcomes, we sought to identify the *most* effective ways to connect zoo visitors with animals to increase knowledge, positive emotions, empathy, and conservation intent.

In this study, we examined which type of encounter had the greatest impact on zoo visitors by measuring a range of psychological constructs after visitors experienced one of three conditions. Those conditions were: (1) viewing a video in animal care quarters of a caretaker talking about stingrays or elephants and conducting a training and painting session with a stingray or elephant (i.e., VIDEO); (2) viewing an in‐person, live demonstration in animal care quarters with a caretaker talking about stingrays or elephants and conducting a training and painting session with a stingray or elephant (i.e., IN‐PERSON); and (3) viewing an in‐person, live demonstration in animal care quarters with a caretaker talking about stingrays or elephants and conducting a training and painting session with a stingray or elephant, and then having the opportunity to touch a stingray or elephant (i.e., TOUCH) (Table 1).

**Table 1:** Summary of conditions in the study.

Condition	Speaker	Animal presence	Location	See animal training and painting?	Touch animal?
Video	Video of caretaker	Video of animal	Animal care quarters	Yes	No
In‐person	In‐person caretaker	Live animal	Animal care quarters	Yes	No
Touch	In‐person caretaker	Live animal	Animal care quarters	Yes	Yes

Specifically, following Miller et al. (2020), we measured the effects of these different types of interactions on zoo visitor cognition (retention of knowledge), affect (emotional feelings), empathic concern (compassion toward wild counterparts; “conservation caring” (Skibins and Powell 2013)), and conservation intent (interest in getting involved in conservation of wild counterparts) (reviewed in Young et al. 2018). We also compared these measures between those who had an experience with a large, familiar mammal species (Asian elephants, *Elephas maximus*) to those who had an experience with a smaller, lesser‐known fish species (~19 cownose stingrays, *Rhinoptera bonasus*, and one bat ray, *Myliobatis californica*). To our knowledge, this is the first study to parse out the effects of touch and species in animal–visitor interactions in a zoo setting.

We expected greater knowledge retention, more positive affect, greater empathic concern, and greater conservation intent, as follows: TOUCH > IN‐PERSON > VIDEO. We also expected the large, familiar species to have a more positive effect on all response measures compared to the smaller, less‐known species. We predicted that overall, visitors' scores would be highest in the TOUCH conditions and lowest in the VIDEO conditions, and that visitors' scores would be higher in the elephant conditions than in the stingray conditions.

## 2 Materials and Methods

Adapted from Miller et al. (2020).

### 2.1 Data Collection Location

Oklahoma City Zoo and Botanical Garden (OKCZ) is located in Oklahoma City, OK, USA (35.5212° N 97.4724° W). At the time of this study, OKCZ was open 0900‐1700 year‐round to visitors except for Tuesdays and Wednesdays from December 7, 2021 to February 9, 2022, Thanksgiving, and Christmas.

### 2.2 Experimental Design and Data Collection

#### 2.2.1 Subjects

**Figure 1:**
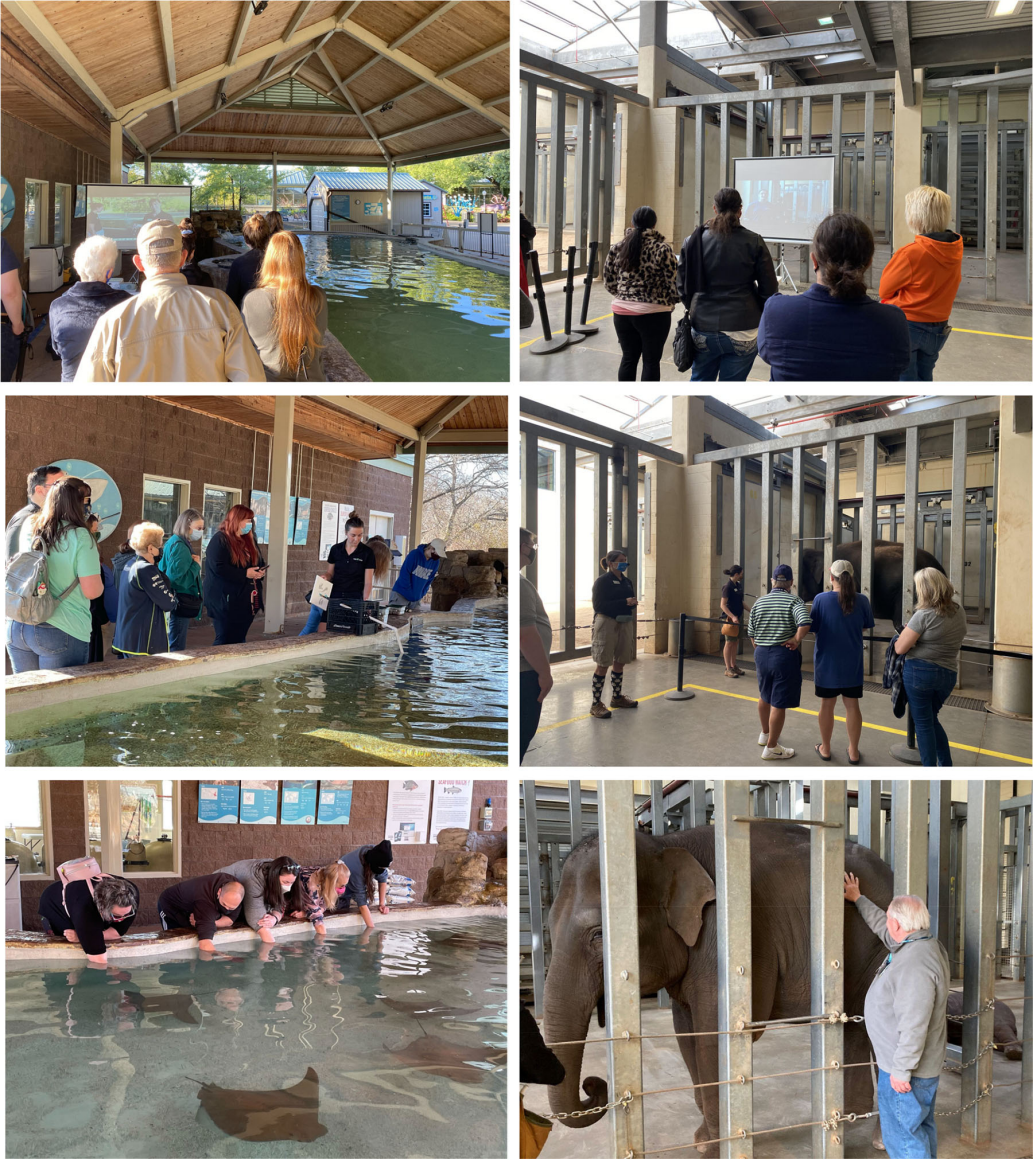
(Left column) Stingray encounters. (Right column) Elephant encounters. (Top row) VIDEO conditions. (Middle row) IN‐PERSON conditions. (Bottom row) TOUCH conditions.

Subjects were 300 members of OKCZ, ranging in age from 18 to 82 years. Participants were recruited using two email messages sent to ~1000 members asking current members 18 years of age or older to volunteer to evaluate a behind‐the‐scenes experience at OKCZ. Subjects were randomly assigned to one of the six study conditions (i.e., elephant VIDEO, elephant IN‐PERSON, elephant TOUCH, stingray VIDEO, stingray IN‐PERSON, stingray TOUCH) on a first‐come‐first‐serve basis (Figure 1). Subjects were unaware of the research questions or the experimental conditions and did not find out what the experience was beforehand. Each subject only participated one time.

### 2.3 Data Collection

We aimed to hold 5 sessions of each condition with 10 subjects per session for a total of 50 subjects per condition. In total, 15 subjects were invited to each session to try to ensure that we had a minimum of 10 subjects and we capped the maximum number at 13. Despite this, we sometimes had fewer than 10 subjects come to their session (range: 3–13 people). The number of sessions and subjects in each condition was: elephant VIDEO: 5 sessions, 52 subjects; elephant IN‐PERSON: 5 sessions, 42 subjects; elephant TOUCH: 5 sessions, 47 subjects; stingray VIDEO: 5 sessions, 55 subjects; stingray IN‐PERSON: 5 sessions, 54 subjects; stingray TOUCH: 5 sessions, 50 subjects.

We instructed participants not to ask any questions related to their experience until after completion of their final survey, at which point we answered any questions they had, without giving away details about the purpose of the study or the conditions (in case they spoke to a friend who would be participating on a later date). All visitors who came to their scheduled session (whether they were selected to participate or not) received a complimentary ticket to a behind‐the‐scenes animal encounter to be scheduled on a later date.

Before the experience, all subjects completed an eight‐question survey (Supporting Information S3: Appendix, Document 1) used to gauge their predisposition toward animals, nature, and conservation. After the completed surveys were collected, we announced the behind‐the‐scenes species and proceeded to the location.

During VIDEO experiences, no animals were present. Participants watched a video‐recorded presentation projected onto a screen (diagonal 203–304 cm) in which a caretaker provided the following information: (1) individual animal's name, year born at/came to OKCZ, (2) diet at OKCZ, (3) method and reasons for training, (4) species' conservation status and threats in the wild, and (5) explanation of animal painting demonstration. In the VIDEO, participants also watched a training and painting demonstration with one elephant or one stingray, one caretaker narrating, and one trainer (Supporting Information S1 and S2: Videos 1 (stingrays) and 2 (elephants)). IN‐PERSON experiences were the same as VIDEO experiences, except that the caretaker narrating provided the information in‐person, live animals were present, and participants saw the training and painting demonstration live (Figure 1). TOUCH experiences were the same as IN‐PERSON, except participants had an opportunity to touch an animal (Figure 1). For elephant TOUCH experiences, participants were directed to approach the elephant one person at a time and touch the side of the elephant through a barrier while she stood still under stimulus control with a caretaker. For stingray TOUCH experiences, participants were directed to place one hand into the pool up to the elbow and wait for a stingray to brush against their hand. Animals, caretakers, and scripts were consistent across VIDEO, IN‐PERSON, and TOUCH experiences within elephant and within stingray conditions.

After each experience, we escorted participants back to the same room in which they completed the first survey, and we asked them to complete a second survey. The second survey included questions to measure participants' reactions to the experience. Specifically, questions focused on emotional affect, knowledge retention, empathic concern, and conservation intent (Supporting Information S3: Appendix, Document 2). There was also a question about gender with “prefer not to say” as one of the options. Participants were also asked to answer “yes” or “no” to whether they had a previous behind‐the‐scenes experience at OKCZ with stingrays for those in the stingray conditions or elephants for those in the elephant conditions. We used an ID for each participant to connect pre‐ and posttest data without identifying the individual. After we collected completed surveys, we answered questions and distributed tickets for a future behind‐the‐scenes experience.

### 2.4 Ethics Approval

This study was approved by OKCZ's Scientific Review Committee (Project ID #2021‐002). Participation was voluntary for all subjects, and subjects had the option to withdraw at any point. No identifying information was collected from participants.

### 2.5 Statistical Analyses

Following Miller et al. (2020), we used *χ* ^2^ tests of independence to assess whether demographic variables differed across treatment groups. We next averaged responses across questions to create composite scores for each visitor, so that each visitor had a predisposition score, an emotion composite score (with fear and sadness reverse‐coded), an empathic concern composite score, and a behavioral intentions composite score (Miller et al. 2020). We summed the total number of correct knowledge‐based questions and divided them by 12 total questions to obtain the percentage correct.

We used generalized linear models to determine if there was an effect of condition and/or species overall on each response—emotion, empathic concern, and behavioral intentions composite score, as well as percentage of knowledge questions correct (beta distribution)—by including condition and species as additive fixed effects. Next, to examine the effect of interactions between condition and species, we used generalized linear mixed models for each response and an interaction between Condition (VIDEO, IN‐PERSON, TOUCH) and Species (elephant, stingray) as a fixed effect, switching the reference level of Condition to conduct multiple comparisons. We also included zoo predisposition score as a fixed effect in our models (Miller et al. 2020). We examined residuals for normality. All analyses were conducted in R (R Core Team 2017).

## 3 Results

There were 300 total participants (52 in elephant VIDEO, 42 in elephant IN‐PERSON, 47 in elephant TOUCH; 55 in stingray VIDEO, 54 in stingray IN‐PERSON, and 50 in stingray TOUCH). We found that there was no association between gender and condition (considering male vs. female, *χ* ^2^ = 5.859, *p* = 0.320; Table 2), ethnicity and condition (considering white vs. native American vs. multiethnic heritage, *χ* ^2^ = 8.093, *p* = 0.6192; Table 2), or level of education and condition (considering some college vs. bachelor's degree vs. some graduate school, *χ* ^2^ = 10.364, *p* = 0.409; Table 2).

**Table 2:** Demographic information (in percentages) for each condition.

Characteristic	Condition					
	Video		In‐person Without Touch		In‐person With Touch	
	E	S	E	S	E	S
Gender						
Male	13.5	30.9	26.2	18.5	17.0	22.0
Female	84.6	69.1	73.8	77.8	80.9	78.0
Other	1.9	0	0	0	0	0
Prefer not to say	0	0	0	1.9	0	0
Ethnicity						
Black	1.9	1.8	0	0	2.1	0
Asian/Pacific Islander	0	0	0	0	2.1	0
White (non‐Hispanic)	73.1	90.9	90.5	81.5	76.6	80.0
Hispanic/Latino	1.9	0	4.8	1.9	2.1	4.0
Native American	3.8	1.8	0	5.6	4.3	6.0
Multiethnic heritage	13.5	5.5	4.8	7.4	8.5	6.0
Prefer not to say	3.8	0	0	1.9	2.1	2.0
Level of education						
Some high school	1.9	1.8	0	0	2.1	0
High school graduate	0	0	0	0	2.1	0
Some college or trade/business school	73.1	90.9	90.5	81.5	76.6	80.0
2‐year associate's degree	1.9	0	4.7	1.9	2.1	4.0
4‐year bachelor's degree	3.8	1.8	0	5.6	4.3	6.0
Some graduate school	13.5	5.5	4.8	7.4	8.5	6.0
Graduate degree	3.8	0	0	1.9	2.1	2.0

*Note:* Blanks were omitted from the analysis.

Abbreviations: E = elephant, S = stingray.

**Figure 2:**
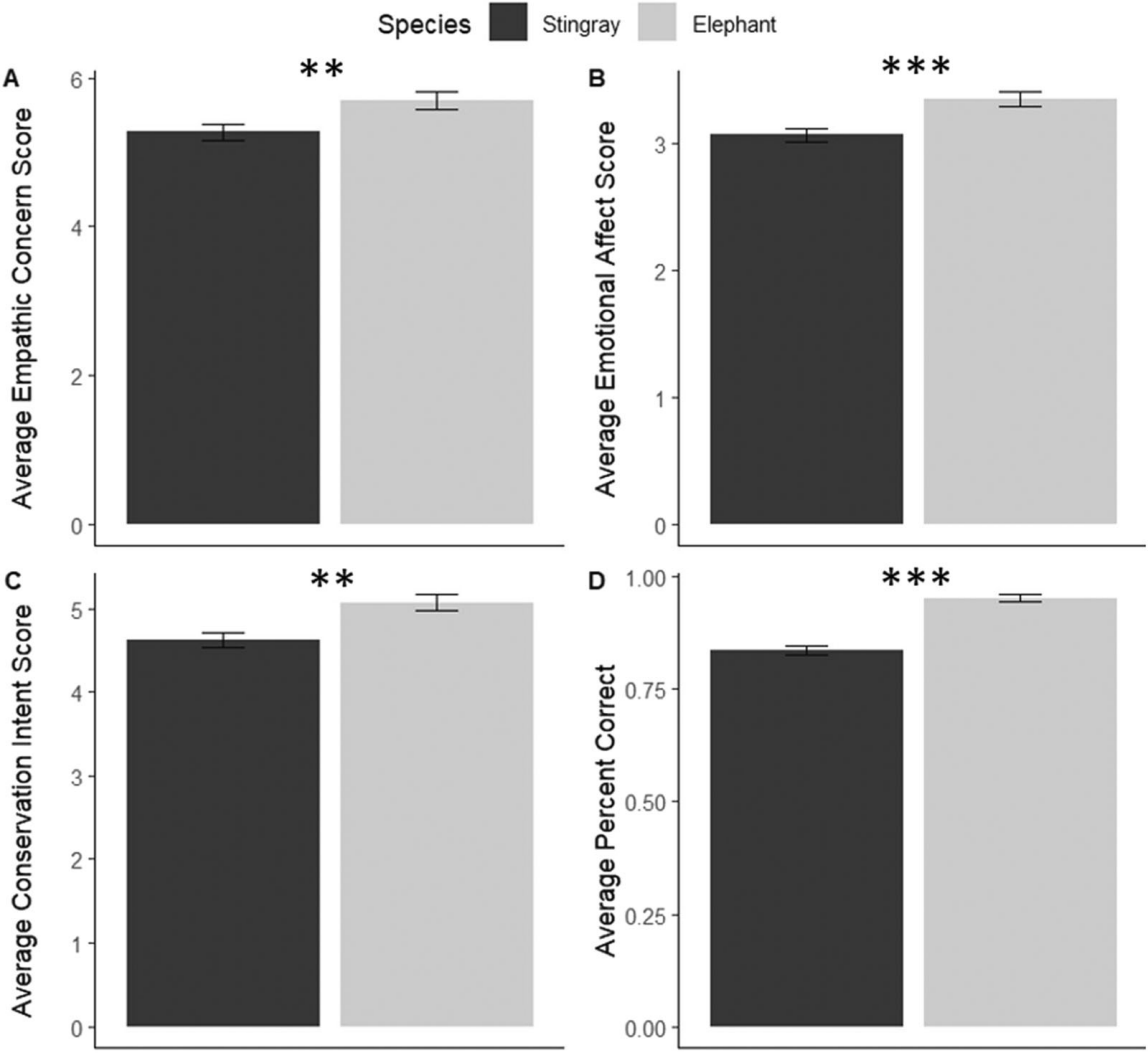
Comparing Species means ± standard error of (A) Empathic Concern Score, (B) Emotional Affect Score, (C) Conservation Intent Score, and (D) Percent Correct. Within each response measure, asterisks denote significant (** = *p* < 0.001, *** = *p* < 0.0001) differences.

We first determined if there was a main effect of species, condition, and/or zoo predisposition as predictors on our response measures. We found a significant effect of species for all measures, in which stingray participants had lower proportions of correct responses (*β* = −1.458, *p* < 0.0001), emotional affect (*β* = −0.286, *p* < 0.0001), empathic concern (*β* = −0.410, *p* = 0.003), and conservation intent scores compared to elephant participants (*β* = −0.433, *p* = 0.0002) (Figure 2).

**Figure 3:**
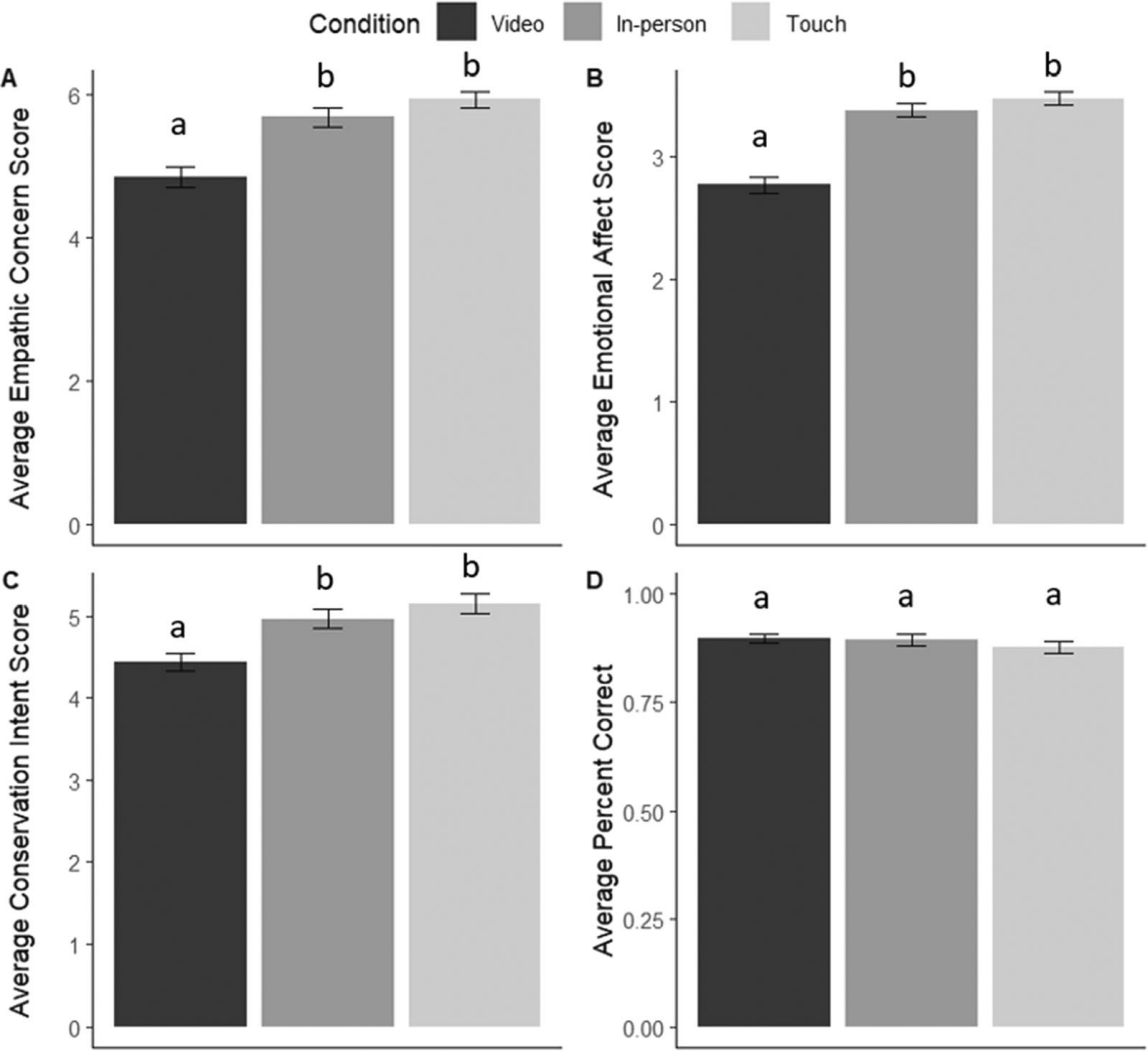
Comparing Condition means ± standard error of (A) Empathic Concern Score, (B) Emotional Affect Score, (C) Conservation Intent Score, and (D) Percent Correct. Within each response measure, a common letter denotes nonsignificant differences between Conditions.

We also found an effect of condition for most measures. With regard to emotional affect score, participants in the VIDEO conditions were significantly lower than participants in the TOUCH conditions (*β* = −0.666, *p* < 0.0001; Figure 3) and participants in the IN‐PERSON conditions (*β* = −0.722, *p* < 0.0001), but there was no significant difference between IN‐PERSON and TOUCH conditions (*p* = 0.316). VIDEO participants (*β* = −0.959, *p* < 0.0001) had lower empathic concern than TOUCH participants and IN‐PERSON participants (*β* = −0.889, *p* < 0.001), but there was no difference for IN‐PERSON participants compared to TOUCH participants (*p* = 0.192). VIDEO participants had lower conservation intent than TOUCH participants (*β* = −0.563, *p* < 0.0001) and IN‐PERSON participants (*β* = −0.694, *p* = 0.002), but there was no difference between IN‐PERSON and TOUCH participants' conservation intent (*p* = 0.295). There was no effect of condition on percentage correct for knowledge questions (*p* = 0.556).

Zoo predisposition was related to most measures. Participants with higher zoo predisposition scores had higher emotional affect scores (*β* = 0.180, *p* = 0.0002), empathic concern (*β* = 0.624, *p* < 0.0001), and conservation intent scores (*β* = 0.663, *p* < 0.001). There was no effect of zoo predisposition on the percentage correct of knowledge questions (*p* = 0.560).

Last, we further explored the interaction between condition and species. None of the interactions were statistically significant (see Tables 3 and 4). Therefore, the main effects of species persisted regardless of condition, and the main effects of condition were regardless of species. Even after a conservative endpoint adjustment (i.e., Bonferroni correction, *p* < 0.007), our significant findings detailed above remained significant.

**Table 3:** Beta estimates, 95% confidence intervals, and *p* values for Species, Condition, and Species*Condition.

	Species	Condition		Species*Condition	
	Stingray	Video	In‐person Without Touch	Stingray*Video	Stingray*In‐person Without Touch
Percent Correct on Questions					
*β*	−1.334	0.208	0.343	−0.215	−0.181
95% CI	−0.917, −1.333	−0.200, 0.617	−0.099, 0.776	−0.782, 0.352	−0.767, 0.406
*p*	*< 0.0001*	0.317	0.120	0.457	0.546
Emotional Affect Composite Score					
*β*	−0.323	−0.774	−0.052	0.131	−0.051
95% CI	−0.548, −0.098	−0.910, −0.552	−0.287, −0.183	−0.180, 0.441	−0.371, 0.269
*p*	*0.005*	*< 0.0001*	0.665	0.410	0.755
Empathic Concern Composite Score					
*β*	−0.517	−1.167	−0.278	0.150	0.088
95% CI	−1.019, −0.014	−1.668, −0.667	−0.803, 0.247	−0.544, 0.844	−0.627, 0.803
*p*	*0.045*	*< 0.0001*	0.301	0.672	0.810
Conservation Intent Composite Score					
*β*	−0.502	−0.811	−0.116	0.197	−0.076
95% CI	−0.940, −0.064	−1.245, −0.377	−0.574, 0.341	−0.406, 0.801	−0.699, 0.547
*p*	*0.025*	*< 0.001*	0.618	0.522	0.811

*Note:* Reference condition group is TOUCH, and reference species group is Elephant. Significant (*p* < 0.05) effects are in italics.

**Table 4:** Reference condition group is IN‐PERSON, and reference species group is Elephant.

	Species	Condition		Species*Condition	
	Stingray	Video	In‐person With Touch	Stingray*Video	Stingray*In‐person With Touch
Percent Correct on Questions					
*β*	−1.514	−0.135	−0.343	−0.035	0.181
95% CI	−1.936, −1.092	0.285, −0.135	0.089, −0.343	−0.606, 0.536	−0.406, 0.767
*p*	*< 0.0001*	0.528	0.120	0.905	0.546
Emotional Affect Composite Score					
*β*	−0.374	−0.722	0.052	0.182	0.051
95% CI	−0.602, −0.147	−0.952, −0.493	−0.183, 0.287	−0.131, 0.494	−0.269, 0.371
*p*	*0.001*	*< 0.0001*	0.665	0.255	0.755
Empathic Concern Composite Score					
*β*	−0.429	−0.889	0.228	0.062	−0.088
95% CI	−0.937, 0.080	−1.402, −0.376	−0.247, 0.803	−0.636, 0.760	−0.803, 0.627
*p*	*0.010*	*< 0.001*	0.301	0.862	0.810
Conservation Intent Composite Score					
*β*	−0.578	−0.694	0.116	0.273	0.076
95% CI	−1.021, −0.135	−1.139, −0.249	−0.341, 0.574	−0.334, 0.880	−0.547, 0.699
*p*	*0.011*	*0.002*	0.618	0.379	0.811

*Note:* Significant (*p* < 0.05) effects are in italics.

## 4 Discussion

To determine the best ways to foster visitor connection with animals in a zoo setting, we examined how different types of experiences with stingrays and elephants influenced visitors' knowledge, emotional affect, empathic concern, and conservation intent. We were specifically interested in two main factors: (1) whether the modality of the experience (i.e., video only, live in‐person without touch, and live in‐person with touch) affected visitor responses, and (2) whether the type of animal involved in the experience affected visitor responses.

We found that the type of experience significantly affected all response measures. Specifically, compared to the IN‐PERSON and TOUCH participants, VIDEO participants exhibited the lowest percent correct on knowledge questions, and also had the lowest scores for emotional affect, empathic concern, and conservation intent. This partially supports previous findings that closer proximity to animals produces pro‐conservation behavioral intentions among visitors (Skibins et al. 2013, 2017). Specifically, IN‐PERSON animal–visitor interactions are more effective than VIDEO‐recorded animal–visitor interactions at producing higher probability of answering questions correctly, producing a positive emotional experience, having greater empathic concern, and exciting visitors to get involved in conservation (Miller et al. 2020). With a larger sample size, it would be interesting to test whether it may be more difficult to absorb information provided during TOUCH experiences compared to IN‐PERSON—or especially VIDEO— experiences (e.g., Whitehouse‐Tedd et al. 2022). Contrary to our findings, other studies found no difference in knowledge gained between visitors who viewed a live animal presentation and those who viewed a video‐recorded presentation, even when a live animal (cheetah, penguin, tortoise, or sloth) was present during the video (Fischer et al. 2024). This raises interesting questions about how the species involved may influence knowledge retention (i.e., George and Cole 2018), and whether the presence of a visible, live animal during the VIDEO condition might alter our results.

The lack of significant differences in participant outcomes between the TOUCH and IN‐PERSON animal conditions suggests zoo visitors need not physically touch animals to experience the benefits that zoos aim to provide—benefits that often come with a fee to visitors, require additional time from caretakers, and may raise welfare concerns. This partially confirms previous work with other species, including Humboldt penguins which visitors could hand feed (Clifford‐Clarke et al. 2022) and cheetahs which visitors could stroke (Whitehouse‐Tedd et al. 2022). We recognize, however, that for many visitors, the opportunity to touch animals is a tremendous draw (e.g., Lacinak 2024), and there may be benefits to TOUCH versus IN‐PERSON animal encounters that we did not measure. Touch experiences, for example, may be important to offer to visitors to help them connect with lesser‐known species. We recommend further research into the outcomes associated with touch animal encounters. Zoo educators could use this knowledge to inform decisions about presentation format and species depending on the educators' targeted outcomes.

Based on previous research (Carr 2016; Colléony et al. 2017; Young et al. 2018;), we predicted that visitors who experienced Asian elephants—being a large and familiar species—would have higher responses across all measures compared to those who experienced stingrays, which are smaller and less familiar. As expected, we found that stingray participants had lower knowledge retention, emotional affect, empathic concern, and conservation intent compared to elephant participants. Other work has also shown that zoo visitors are more interested in mammals (Carr 2016), especially Asian elephants (Moss and Esson 2010) and other eye‐catching large mammals (Landová et al. 2018), which can increase outcomes such as empathy (Young et al. 2018) and pro‐conservation behaviors (Skibins and Powell 2013). Given zoo visitors' preference for large charismatic mammals (with which visitors can connect more easily given that they share humans' same environment, unlike aquatic species), it is unsurprising that zoos' animal collections are biased toward large mammals that people perceive as attractive (Frynta et al. 2013).

Additionally, charisma has been recognized as a driving factor that often outweighs conservation needs when zoos and nongovernmental organizations decide which species are the focus of their conservation efforts (Krause and Robinson 2017). This has led some to advocate for better “marketing” for less liked species/taxa (i.e., insects) that are undergoing significant population decline and receive less conservation investment than charismatic megafauna (Hart and Sumner 2020). A key aspect of that marketing approach is effective promotion (i.e., tailoring the message to engage the audience) (Hart and Sumner 2020). Our findings indicate that in‐person experiences involving opportunities to hear directly from an animal's caretaker are especially important to increase people's caring and conservation intent for underappreciated animals. Although responses on all measures were lower for stingrays than elephants, in‐person experiences and those involving touch opportunities did increase participants' responses across all measures for stingrays.

There are caveats to interpreting our findings. First, we recruited zoo members because they were easily accessible, but they are also likely more conservation motivated than a nonmember. Anecdotally, moreover, experience might have slightly differed within each condition from day to day based on a number of factors. For example, we noticed differences in visitor responses/potential disruption: Some people reacted to the videos (e.g., “Wow! Look, she's painting!”), while in other sessions, there were no noticeable reactions. Also, elephant groups received a golf cart ride to and from the elephant care quarters, whereas stingray groups walked to the stingray pool. This could have affected participants' impressions of their overall experience since elephant participants could potentially see more of the zoo and chat more amongst themselves. Although we tried to ensure that each group had an equal number of participants by inviting more people than would be needed in case of cancellations, we still had sessions in which there were fewer than 10 participants (i.e., due to “no‐shows”), which could also have affected the visitors' perceptions of the encounter.

We also noticed that visitors talked excitedly after the in‐person (both TOUCH and IN‐PERSON) elephant encounters and were less excited after the video experiences for both species. Future studies could collect qualitative data (e.g., adjectives visitors verbalized) as an additional response measure. Moreover, stingrays were more difficult to view clearly compared to elephants, because the stingrays were swimming underwater. Additionally, not all participants in the stingray TOUCH condition were able to touch a stingray, because some stingrays did not choose to brush against the hands of all participants, whereas the elephant was trained to participate in touching using operant conditioning and chose to participate in all TOUCH sessions. Therefore, it may have been more difficult for participants to connect with stingrays compared to elephants. Since we cannot rule out these potential confounds, it would be worthwhile for future studies to test differences in the constructs we measured after experiences with species of similar sizes and viewability. Finally, although our caretaker script included conservation threats, it did not include calls to action; this is a key component of encouraging conservation behavior among visitors (e.g., Skibins et al. 2017) and should be embedded in future caretaker talks.

Our results highlight important effects of behind‐the‐scenes encounters on zoo visitors. In the face of burgeoning online videos, robotics, artificial intelligence, and augmented reality development (e.g., animatronic “robot dolphins,” Edge Innovations: https://www.edgefx.com/real-time-animatronics; hologram zoo, Scollen and Mason 2024), we found that zoos continue to serve an important role in connecting humans with nonhuman animals. Moreover, our findings support previous research in which zoo visitors report feeling connected to nature (Bruni et al. 2008) after viewing live animals and express interest in helping wild animals (Collins et al. 2021; Hayward and Rothenberg 2004; Miller et al. 2020; Priestley et al. 2023), indicating that zoo animals can encourage conservation of their wild counterparts. Notably, Schuler and Skibins (2023) found few significant differences in the pro‐conservation behavior of tourists who viewed animals in human care versus wild animals, providing further evidence that zoo settings are effective in eliciting conservation intent. We sought to expand understanding about which species and types of experience can have the greatest positive impact on zoo visitors, but further research is needed. Specifically, investigating the influence of an individual animal's characteristics (i.e., sex, age, personality, life story) on creating meaningful animal–visitor interaction/connection would be useful. Additionally, there could be benefits associated with touching zoo animals depending on the characteristics of zoo visitors (e.g., children, vision impaired, neurodivergent) that we did not capture.

## Supporting information


**Video 1.** Video used for video condition with stingray participants.

**Video 2.** Video used for video condition with elephant participants.

APPENDIX.
Document 1. Presurvey for elephant participants.
Document 2. Postsurvey for elephant participants.

## Data Availability

The data that support the findings of this study are available from the corresponding author upon reasonable request.
