# Supplementary material for: Impact of Animal Encounter Modality and Species on Zoo Visitor Knowledge, Concern, and Conservation Intent
Source: Zoo Biol. 2025 Sep 15;44(6):652–61. doi: 10.1002/zoo.70023 (PMC12681049; doi:10.1002/zoo.70023)
Supplement: Supplementary file 3 — APPENDIX. Document 1. Presurvey for elephant participants. Document 2. Postsurvey for elephant participants. [file ZOO-44-652-s001.docx]

APPENDIX

Document 1. Pre-survey for elephant participants

**Survey 1E - Oklahoma City Zoo**

**Birth Date: _____ - _____ - ________**

**Rate how well each statement describes you from “does not describe me at all” to “describes me perfectly”.**

|  | **Does Not Describe Me At All** | **Describes Me Slightly** | **Describes Me Moderately** | **Describes Me  Well** | **Describes Me Perfectly** |
| --- | --- | --- | --- | --- | --- |
| 1. I am ordinarily interested in animals. | O | O | O | O | O |
| 1. I often feel a sense of connection with nature. | O | O | O | O | O |
| 1. I have a good understanding of wildlife conservation issues. | O | O | O | O | O |
| 1. I pay attention to news about environmental issues. | O | O | O | O | O |
| 1. I usually try to help protect and preserve local wildlife habitats. | O | O | O | O | O |
| 1. I tend to support conservation organizations (volunteer my time, make a donation, sign a petition, etc.) | O | O | O | O | O |
| 1. I typically engage in conservation efforts during my daily activities (recycling, reducing energy use, buying earth-friendly products). | O | O | O | O | O |
| 1. I spend as much time as I can in natural settings such as woods, prairies, mountains, or lakes. | O | O | O | O | O |

Document 2. Post-survey for elephant participants.

For Administrator Use:

1_____ 2_____ 3_____

**Survey 2E - Oklahoma City Zoo**

**Birth Date: _____ - _____ - ________**

**Please rate the appropriateness of the following words for describing your emotions during the experience, from “not appropriate at all” to “very appropriate”.**

|  | **Not Appropriate At All** | **Slightly Inappropriate** | **Neither Appropriate nor Inappropriate** | **Slightly Appropriate** | **Very Appropriate** |
| --- | --- | --- | --- | --- | --- |
| 1. Awe | O | O | O | O | O |
| 1. Contentment | O | O | O | O | O |
| 1. Excitement | O | O | O | O | O |
| 1. Fear | O | O | O | O | O |
| 1. Joy | O | O | O | O | O |
| 1. Love | O | O | O | O | O |
| 1. Pride | O | O | O | O | O |
| 1. Amusement | O | O | O | O | O |
| 1. Sadness | O | O | O | O | O |
| 1. Surprise | O | O | O | O | O |

**Multiple Choice: Circle one choice for each question**

1. What year was Achara born?
2. 2021
3. 1998
4. 1989
5. 2014
6. The reason for training Achara is for…
7. Providing her with care and exercise
8. Our fun and entertainment
9. Keeping up with what the other zoos do
10. None of the above
11. Achara is trained…
12. Using negative reinforcement
13. Use positive reinforcement
14. By accident
15. By watching the other elephants
16. A clicking sound is used to let Achara know…
17. What time it is
18. She has to try harder
19. There is no more food left
20. She did the behavior correctly
21. How many species of elephants are found around the world?
22. 1
23. 2
24. 3
25. 4

For Administrator Use:

1_____ 2_____ 3_____

1. Asian elephants are classified as…
2. Gray
3. Extinct
4. Endangered
5. Abundant
6. Which of the following exhibit features is designed to increase the amount of time Achara spends exercising?
7. Swimming pool
8. Large hill for climbing
9. Tall feeders for reaching
10. All of the above
11. How does Achara paint?
12. With her trunk
13. With her feet
14. With her mouth
15. She can’t paint
16. What does Achara receive during training?
17. Pats on the head
18. Preferred fruits and vegetables
19. Leaves
20. Hay
21. What is the number one threat to Asian elephants in the wild?
22. Disease
23. Habitat loss
24. Hunting
25. Lions
26. The elephant is considered a(n)…
27. Herbivore
28. Carnivore
29. Omnivore
30. None of the above

**What were your overall reactions to the experience, from “completely false” to “completely true”.**

|  | **Completely False** | **Mostly False** | **Slightly False** | **Neutral** | **Somewhat True** | **Mostly True** | **Completely True** |
| --- | --- | --- | --- | --- | --- | --- | --- |
| 1. I have a greater sense of my connection with nature because of this experience. | O | O | O | O | O | O | O |
| 1. This experience has made me more concerned about the well-being of these animals in the wild. | O | O | O | O | O | O | O |

**Thinking about your overall reactions to the experience, how much do you agree with the following statements from “strongly disagree” to “strongly agree”?**

|  | **Strongly Disagree** | **Mostly Disagree** | **Somewhat Disagree** | **Neither Agree or Disagree** | **Somewhat Agree** | **Mostly Agree** | **Strongly Agree** |
| --- | --- | --- | --- | --- | --- | --- | --- |
| 1. I want to spend more time learning about elephants (reading books, searching the Internet, etc.) | O | O | O | O | O | O | O |
| 1. I am going to have discussions with others about elephants (discuss conservation issues, what they can do to help, etc.) | O | O | O | O | O | O | O |
| 1. I now have a better understanding of what actions I can take that will help protect and preserve elephants and their habitats. | O | O | O | O | O | O | O |
| 1. As a result of this experience, I want to donate money to an elephant conservation organization. | O | O | O | O | O | O | O |

**Gender:** _______Female _______Male _______Other _______Prefer Not to Say

**Have you participated in an elephant behind-the-scenes experience at OKC Zoo before today?** ______Yes ______No

**Ethnicity:**

_______Black/African American

_______Asian/Pacific Islander

_______White (non-Hispanic)

_______Hispanic/Latino

_______Native American

_______Multi-Ethnic Heritage

_______Prefer Not to Say

**Highest Level of Education:**

_______Some High School

_______High School Graduate

_______Some College or Trade/Business School

_______2-Year Associate’s Degree

_______4-Year Bachelor’s Degree

_______Some Graduate School

_______Graduate Degree
